# CDGSH iron-sulfur domain 2 as a therapeutic target for stroke: an opinion article

**DOI:** 10.3389/fnmol.2024.1386118

**Published:** 2024-05-28

**Authors:** Chao-Kang Feng, Wei-Jung Chen, Woon-Man Kung, Yu-Yo Sun, Muh-Shi Lin

**Affiliations:** ^1^Department of Nursing, College of Nursing, Hung Kuang University, Taichung, Taiwan; ^2^Department of Biotechnology and Animal Science, College of Bioresources, National Ilan University, Yilan, Taiwan; ^3^Division of Neurosurgery, Department of Surgery, Taipei Tzu Chi Hospital, Buddhist Tzu Chi Medical Foundation, New Taipei City, Taiwan; ^4^Department of Exercise and Health Promotion, College of Kinesiology and Health, Chinese Culture University, Taipei, Taiwan; ^5^Institute of Biopharmaceutical Sciences, National Sun Yat-sen University, Kaohsiung, Taiwan; ^6^Department of Neuroscience, Center for Brain Immunology and Glia (BIG), University of Virginia School of Medicine, Charlottesville, VA, United States; ^7^Department of Health Business Administration, College of Medical and Health Care, Hung Kuang University, Taichung, Taiwan; ^8^Division of Neurosurgery, Department of Surgery, Kuang Tien General Hospital, Taichung, Taiwan

**Keywords:** CISD2, anti-inflammation, CISD2 as NF-κB antagonist, CISD2 targeting stroke, neurological injury and disease

## Introduction

Dramatic changes in global climate and metabolic dysfunction in the population, attributed to dietary modifications, have rendered stroke a significant threat to human health. Within the neuron-rich environment of the brain, including immune cells and neural circuits, the embolization or rupture of a cerebral blood vessel with bleeding inevitably triggers two major pathological mechanisms: dysfunctional mitochondria and neuroinflammatory response. These mechanisms cause a vicious cycle of compounding effects on each other, featuring increased blood–brain barrier (BBB) permeability (rendering the brain no longer immune-privileged upon its disruption), mitochondrial reactive oxygen species (ROS) production, detrimental activation of glial cells, neurocircuitry dysfunction, and ultimately, neuronal apoptosis. Although neurosurgery and vascular interventions can effectively address the primary insults following a stroke, such as minimally invasive removal of *in situ* hematoma or the clearance of intravascular thrombi, they remain incapable of eliminating profound secondary insults at the molecular level, as described above. The prognosis of stroke is influenced by distinct eloquent areas of the brain. The potential to reduce cerebral inflammation, restore mitochondrial antioxidant and energogenic functions, promote neuroconnectivity, and even regain neural circuit transmission, will hopefully minimize the sequelae of stroke.

## CISD2—the second member of the NEET protein family in humans

The NEET family of proteins is named after the unique amino acid sequence-asparagine-glutamic acid-glutamic acid-threonine [Asn-Glu-Glu-Thr (N-E-E-T)]-found at the C-terminus of each family member. A common feature of the NEET protein family is the presence of a particular sequence motif, CDGSH, which constitutes the so-called CDGSH iron-sulfur structural domain. This domain comprises 3-cysteine (Cys)-1-histidine (His) linked to the [2Fe-2S] cluster within the CDGSH structural domain. Consequently, by incorporating a distinctive CDGSH domain combined with [2Fe-2S] clusters, the NEET protein family plays a pivotal role in redox reactions and electron transport.

In humans, the NEET family includes three members, CISD1, CISD2, and CISD3, named for the CDGSH iron-sulfur domain. The second member, the CISD2 protein, is encoded by the CISD2 gene (located at “q24” on the long arm of chromosome 4) (Kung, 2021a). As an iron-sulfur and structural protein residing in the mitochondria, endoplasmic reticulum (ER), and mitochondria-associated ER membranes (MAMs), CISD2 acts as a transport conduit. Through CISD2 family associations, it facilitates redox reactions, [Fe-S] cluster transfer, and enhances the transportation of labile iron between the mitochondria and cytoplasm, thereby impeding mitochondrial Fe/ROS/Fe-S imbalance, ROS toxicity, and mitochondrial Fe excess. Importantly, CISD2 dominates the regulation of upstream pathways of neuroinflammation and acts as an antagonist of NF-κB (Lin, 2020), thereby extending its protective effects to anti-inflammation and the preservation of mitochondrial function. These actions constitute two of the key causative mechanisms of neurological injury and disease.

## CISD2 depletion by neurological injury and disease is detrimental

In neurological insults, neurodegenerative conditions such as spinal cord injury (Lin et al., 2015), or aging/neurodegenerative clinical conditions (Lin et al., 2019), putative factors such as structural damage from external forces, ER stress, or potentially relevant unfolded protein response (UPR) (Kung et al., 2022) can lead to CISD2 protein degradation, allowing for deleterious pro-inflammatory and mitochondrial dysfunction. Specifically, CISD2 expression was observed to be downregulated in animals after spinal cord hemisection: in mice, at 104 weeks of age; and in astrocytes, after a prolonged culture (35 DIV). In a CISD2 knockdown model using siCISD2, the deletion of CISD2 resulted in a significant increase in iNOS generation and mitochondrial dysfunction, including decreased mitochondrial membrane potential, enhanced ROS release, and, ultimately, apoptosis.

## A similar trend toward a decrease in CISD2 after stroke

Regarding stroke applications, consistent with previous observations, CISD2 tends to decline in animals with middle cerebral artery occlusion (MCAO)-induced stroke and in oxygen-glucose deprivation/reoxygenation (OGD/R)-induced hippocampal neuron injury, with a subsequent detrimental effect on the outcome of stroke.

Because CISD2 trends downward in a similar manner after stroke, protective strategies are required to increase CISD2 expression. Strategies to enhance CISD2, such as overexpression, cryogen spray cooling, and application of the phytochemical curcumin or wild bitter melon, can be beneficial for neurological injury or disease (Kung, 2021b). In stroke, CISD2-overexpression greatly ameliorated ischemia-associated mitochondrial dysfunction and subsequent apoptosis (Fan et al., 2022; Ren et al., 2022; Hu et al., 2023).

## Pitfalls and clinical considerations for cancer

Notably, the aforementioned CISD2-elevating strategy in injury-abolished CISD2 conditions is irrelevant and may even be contraindicated in carcinomatous subjects. Unlike damaged or degenerated neural tissues, cancer cells exhibit abnormally high CISD2 expression, and CISD2 overexpression in these cells result in a significantly lower overall survival rate (Li et al., 2017; Sun et al., 2017; Shao et al., 2021; Zhang et al., 2022). Conversely, attenuating CISD2 in this state inhibits cancer cell proliferation and invasion, and provides a better prognosis (Sun et al., 2017; Shao et al., 2021).

## Special attention to CISD2 overexpression in cryptogenic stroke

Cancer and cerebrovascular disease may be interrelated owing to common susceptibility factors (such as, demographic characteristics of older adults and angiographic variables). Thus, patients with stroke of unknown etiology or origin develop cryptogenic malignancies to an increasing extent. Nearly 20% of patients with cryptogenic stroke develop occult malignancies at onset (Salazar-Camelo et al., 2021). Rigorous cancer screening and long-term cancer surveillance are recommended for such patients. In the management of patients with stroke, especially those with a cryptogenic variant, excessive care must be taken when applying CISD2-based policies, particularly those targeting CISD2 overexpression. For the remaining 80% of patients with cryptogenic stroke without cancer, long-term monitoring for cancer is recommended under the CISD2-based policy.

## Brief outlook

There might be a significant increase in the number of individuals affected by stroke with the aging population. With the physical dysfunction in the population, this will likely increase the burden on society. Stroke is characterized by two major pathological mechanisms: mitochondrial dysfunction and neuroinflammatory response, which ultimately contribute to neuronal apoptosis and neurological sequelae.

CISD2 functions include calcium metabolism, apoptosis, and longevity. As a member of the NEET family, CISD2 possesses a [2Fe-2S] cluster, which regulates apoptosis-related functions and mitochondrial Fe/ROS/Fe-S homeostasis. Acting as an NF-κB antagonist, CISD2 provides a rationale for the treatment of the two NF-κB-induced pathomechanisms that lead to stroke. New strategies targeting the enhancement of CISD2 hold promise for the treatment of stroke, central nervous system injuries, and neurodegenerative diseases. However, CISD2 overexpression may exacerbate cancer progression and must be considered in clinical applications. Nevertheless, strokes of cryptogenic origin are associated with occult malignancies. Thus, it is recommended that patients with a stroke of unknown etiology or origin should undergo aggressive cancer screening and long-term cancer surveillance.

## Author contributions

C-KF: Writing – original draft. W-JC: Conceptualization, Writing – original draft. W-MK: Data curation, Writing – original draft. Y-YS: Data curation, Writing – original draft. M-SL: Writing – review & editing.
